# Comparing Psychopathological Symptoms in Portuguese Football Fans and Non-fans

**DOI:** 10.3390/bs10050085

**Published:** 2020-05-01

**Authors:** Ângela Leite, Ana Ramires, Rui Costa, Filipa Castro, Hélder Fernando Pedrosa e Sousa, Diogo Guedes Vidal, Maria Alzira Pimenta Dinis

**Affiliations:** 1 Faculty of Philosophy and Social Sciences, Portuguese Catholic University, Rua de Camões 60, 4710-362 Braga, Portugal; angelamtleite@gmail.com; 2 Tourism & Hospitality School, University Europeia, Rua Laura Ayres 4, 1650-510 Lisboa, Portugal; ana.ramires.ps@gmail.com; 3 Sports & Health School, University Europeia, Estrada da Correia 53, 1500-210 Lisboa, Portugal; ruialexx@gmail.com; 4 Faculty of Psychology, Education and Sport, University Lusófona, Rua de Augusto Rosa 24, 4000-098 Porto, Portugal; lipa.castro@hotmail.com; 5 Department of Mathematics (DM. UTAD), University of Trás-os-Montes and Alto Douro, Quinta de Prados, 5001-801 Vila Real, Portugal; hfps@utad.pt; 6 UFP Energy, Environment and Health Research Unit (FP-ENAS), University Fernando Pessoa (UFP), Praça 9 de Abril 349, 4249-004 Porto, Portugal; madinis@ufp.edu.pt

**Keywords:** violent behavior, football fans and non-fans, psychopathological symptoms, Portuguese population

## Abstract

The present study aims to characterize football fans and non-fans and to compare their psychopathological symptoms with the latest normative values for the Portuguese population from Canavarro in 2007. Results showed that football fans and non-fans are mostly male, have an affective relationship, are childless, have secondary education or a high degree, and are employed or students; fans are more likely to be male, dating, unemployed, to have elementary education and be younger than non-fans. Football fans present significantly higher psychopathological symptoms than non-fans in somatization, interpersonal sensitivity, anxiety, hostility, paranoid ideation and psychoticism and all psychopathological indexes. Football fans present values very close to those of populations with emotional distress in hostility and are above the mean of the general population in obsession–compulsion, hostility, paranoid ideation and psychoticism.

## 1. Introduction

### 1.1. Context

Football is an identity assertion and a sociocultural space [1], and is highly complex, multi-faceted and difficult to define [2]. Football is the most popular spectator sport in the world [3] and, according to the last World Football Report [4], more than 40% of people aged 16 or older in major population centers around the world consider themselves interested or very interested in following football, more than in any other sport. The same report states that the United Arab Emirates tops the list with 80% of the country’s population assumed as fans, followed by Thailand (78%), Chile (75%) and Portugal (75%), where this sport is highly valued. Another interesting fact of this report is that football is a sport transcending gender. In fact, football is the most popular sport among women globally. However, this global crowd phenomenon also carries a history of major incidents of public disorder [5]. Violence associated with football is mostly the responsibility of organized football fans, although some spectators who do not integrate the fan clubs may also be responsible for some of that violence [6]. These violent incidents are sometimes interpreted as the result of hooligans’ behavior, and other times as complex crowd events [7,8,9]. The concept of hooliganism is very wide, encompassing alcoholized fans and situations involving hundreds of fans [5,10]. The hooligan subculture emerged in the 1960s in England, in gangs of skinheads from disadvantaged urban areas where the street culture was associated with riots and the confrontation between rival neighborhoods [8,11]. In the 1970s, the ultra-subculture emerged in Italy, in a political context of struggle and social criticism, having been initially linked to members of extreme-left organizations [12]. Ultras tend to engage in clashes in a team spirit. They usually use the style of urban warfare, although they are less prone to riots than hooligans [1,13].

### 1.2. Predisposing and Predicting Factors

Strang, Baker, Pollard and Hofman [14] studied the violent and antisocial behaviors that take place at football matches and the associated factors. These authors presented antisocial and violent behavior displayed by football fans, namely “hooliganism, verbal and physical aggression, destruction of property, acts of vandalism and theft” [14] (p. 6). Alcohol consumption; rivalry between football fans; unemployment, repression by state agents and ethnic-nationality tensions; spatial factors (e.g., sharing physical space with fans of other teams); situational factors (e.g., the day of the week on which the match is held); and the impact of match play on fan behavior—all of these may be contributing factors to the occurrence of violent and antisocial behavior [14]. Additionally, internal psychological factors (e.g., adrenaline rush generated in individuals by antagonizing rival fans) and external or relational psychological factors (e.g., lack of social support systems) can lead to disorder [14]. However, the reasons for football fans to develop this kind of behavior may be because subjects choosing these fan clubs already have a predisposition to violent physical confrontations [15]. In fact, several authors [16,17,18,19] found similarities in values and behaviors associated with subcultures of fans, particularly regarding the high emotional involvement, the strong identification and affiliation with their clubs, the incorporation of traditional male values of Western culture and the reproduction of a code of honor. Wakefield and Wann [20] identified two groups of highly identified fans: those with low dysfunction, i.e., people who seldom or never complain and rarely, if ever, come into confrontations, and those with a high level of dysfunctionality [21]. When compared with less dysfunctional fans, fans identified as dysfunctional can be characterized as individuals likely to blast with the referees, who believe that alcohol consumption is a necessary activity on a game day, who are critical of the stadium services, and who are frequent consumers of sports media [22,23]. The identification of fans refers to the psychological connection they have with their chosen sports team. Fans highly identified with the team feel less control over their behavior in games than fans with a moderate or low identification. However, neither attitudes related with aggression nor subjective norms on aggression differed depending on the level of identification with the team [24,25]. Vallerand and colleagues [26] (p. 1279) showed that “harmonious passion was positively associated with adaptive behaviors (e.g. celebrate the team’s victory), whereas obsessive passion was positively associated with maladaptive behaviors (e.g., risking losing one’s job to go to a game)”. The reasons to become a fan of a club can be summarized under a taxonomy of essential needs whose satisfaction leads people to turn to sport, becoming fans in order to satisfy them. This taxonomy contains three core needs: validation—the need to confirm or justify oneself and can be satisfied; pleasure—the hedonistic need to experience satisfaction or pleasure and can be satisfied; and excitement—the desire for stimulation and excitement and can be satisfied [27]. Yun-Tsan [28] stated that significant interaction effects exist between spectator motivation and team identification, regarding team loyalty and switching intention. According to Pereira [29], the most common reasons to be a football fan are the affiliation with the group, and aesthetic, entertainment and eustress reasons, while less common reasons are economic and family factors. Men are more motivated about the aesthetic, eustress, entertainment and escape factors when compared to women. The former are more motivated by family reasons when compared to men. Fans seem to feel more motivated from the intrinsic point of view than from the extrinsic point of view [30].

### 1.3. Fans’ Social Characteristics and Psychopathological Symptoms

Traditionally, football fans are predominantly white, male, and belonging to the working class [31], but football has more recently become popular among a broader cross-section of society, especially women [32,33]. Highly dysfunctional fans can be characterized as having less education, lower incomes, and being younger, male, single and living in apartments without children at home [20,34]. According to Jones [32], women experience a tension between fan and gender identities. Although they may have to minimize femininity to be considered real fans, many women feel that they belong to the club despite being women. Wolensky [35] found that men and women did not differ in levels of fandom involvement. However, women demonstrated higher emotional expressivity (positive, negative, impulse strength and overall) and more cognitive distortions. Being female and having higher levels of fan involvement were predictive of cognitive distortions. 

Some studies suggest a connection between fandom and psychopathology. According to Toder-Alon, Icekson and Shuv-Ami [36], older fans reported higher levels of sports fandom and lower levels of self-reported aggression and acceptance of aggression; age moderated the relationships between team identification (or fandom) and self-reported aggression, team identification (or fandom) being more strongly associated with self-reported fan aggression among younger fans than among older fans. Violent groups have more symptoms of phobia, anxiety, depression, obsessive thoughts, somatization, interpersonal sensitivity, aggression, psychosis and self-esteem than non-violent groups, and these morbid symptoms are effective in the incidence of aggression [37]. Fans of football show less activation in brain regions involved in pain perception and empathy (anterior cingulate cortex, fusiform gyrus, insula and temporal pole) when viewing violence in the context of football when compared to more violent images. Non-fans of football showed no such effect and had higher activation levels for the specified brain regions [38]. Heun and Pringle [39] state that playing and watching football games may negatively affect subjective mental health, even though qualitative studies indicate mental health benefits of playing or watching football. Fan dysfunction is positively related to perceptions of the appropriateness of verbal aggression and physical aggression; team identification is unrelated to perceptions of the appropriateness of verbal aggression but negatively related to perceptions of the appropriateness of physical aggression [40]. Courtney and Wann [41] proved that having been a bully as a child predicts level of fan dysfunction as an adult.

## 2. Materials and Methods

### 2.1. Aims

The above-mentioned studies suggest an association between football club fans and violent behavior. Further, they suggest a link between violent behavior and psychopathology. However, there is very little research that directly relates football club fan members and psychopathology. Thus, this study seeks to investigate whether the origin of these more dysfunctional behaviors may lie in psychopathology, the objective of this study being to characterize football club members (fan and non-fans) of Portuguese football teams and to compare their Brief Symptom Inventory (BSI) values with the normative values established by Canavarro [42] for the Portuguese population in 2007. Fan club members belong to an organized group that is supported by the club and whose identity, loyalty and dedication are very high. The non-fans or members of the club are supporters of the club, spectators that accompany the team, but do not belong to any organized group, and their identity, loyalty and dedication are lower. In light of the literature review, the following research hypotheses were formulated:

**Hypothesis 1 (H1).**  *Sociodemographic characteristics of football fans and non-fans are significantly different.*

**Hypothesis 2 (H2).**  *Football fans present significantly higher BSI values than non-fans.*

**Hypothesis 3 (H3).**  *Football fans and non-fans present significant differences in the BSI dimensions and indexes values when compared to the suggested normative values of the BSI Portuguese version established by Canavarro [42] in 2007.*

### 2.2. Measures

Besides the sociodemographic questionnaire, the protocol included the 53 items of BSI by Canavarro [43], which was the Portuguese adaptation of the BSI by Derogatis and Spencer [44]. This inventory assesses psychopathological symptoms in terms of nine dimensions of symptoms and three global indexes, with the latter being summary reviews of emotional disturbance. The nine primary subscales are: somatization, obsession–compulsion, interpersonal sensitivity, depression, anxiety, hostility, phobic anxiety, paranoid ideation, and psychoticism. The three global indexes are: General Symptom Index (GSI), Total Positive Symptom (TPS) and Positive Symptom Index (PSI). For the Portuguese population, Canavarro [42] has established in 2007 (last update), normative values of the subscales and global indexes for the general population and for the population expressing emotional disturbances, which are presented in Table 1. The instrument developed by Canavarro [43], widely used in Portugal in the field of psychopathology, is very robust and has high levels of reliability, as Derrogatis’ original version [44] also has. Additionally, Canavarro [42] sets the 1.7 value as the cut-off point based on the PSI above, to which the authors found emotional disturbance. 

### 2.3. Procedures

After designing the protocol (sociodemographic and BSI questionnaire), club members were informed about the research content and its objectives. Confidentiality and anonymity were guaranteed, and informed consent was obtained. The information regarding the participants’ status (fans or non-fans) was self-reported by participants. The distinction made between fans and non-fans results from the existence of fans that belong to an organized group of supporters that, in most cases, are economically supported by the clubs themselves. These groups accompany the club in all matches and assume the characteristics of the club, namely its music and its symbols, as part of its personal identity. By contrast, non-fans do not belong to these organized clubs; they identify with the club, watch the games and support the club, but do not participate in the group activities to praise and support the club. Both fans and non-fans are members of the club, that is, they pay an annual fee to make it easier to watch the games. Fans have many advantages because they are the ones who always accompany the clubs and support them. In the applied protocol, participants were asked if they belong to these support clubs organized by teams or not.

The questionnaire was applied online, and 1302 members participated voluntarily. Only completed questionnaires, a total of 1166, were considered valid for the study. All procedures followed were in accordance with the ethical standards of the responsible committee on human experimentation (institutional and national) and with the Helsinki Declaration of 1964, as revised in 2013.

### 2.4. Statistical Analysis

Descriptive and inferential statistical procedures were carried out. To analyze the association between the groups and the categorical variables of interest, Chi-square tests were performed, and when results were significant, post hoc z-tests on the adjusted residuals with Bonferroni correction were conducted. Independent samples *t*-tests, with the Bonferroni correction to control the familywise error rate, were used to examine the differences between groups. It is one of the most widely used statistical hypothesis tests [45] to determine whether there is a significant difference between the means of two groups. The effect size measure used was Cohen’s *d*, recommended for independent groups when population variance is unknown [46]. Cronbach’s alpha was applied to assess BSI factors reliability and interpreted according to Kline’s benchmarks [47]. The 0.05 level of significance was set for all data analyses. All statistical analyses were performed using SPSS software, version 25 [48].

## 3. Results

The overall sample consisted of 1166 participants, 407 fan club members (hereafter abbreviated as FCM) and 759 non-fans (hereafter abbreviated as NF) of several Portuguese football teams (*Sport Lisboa Benfica*—38%, *Sporting Clube de Portugal*—26%, *Futebol Clube do Porto*—21%, others—15%). The age ranged from 18 to 85 years, and half of the participants were younger than 24 years. The majority was male (63%), had an affective relationship (48%), did not have children (71%), had a secondary education (44%) or a high degree (41%), and was employed (48%) or was a student (42%). Both subsamples, FCM and NF, share the sociodemographic pattern of the overall sample (Table 2).

Chi-square tests and an independent samples *t*-test were used to access differences between FCM and NF sociodemographic characteristics. The Chi-square tests assumptions were met. Statistically significant associations, but with small effect size, were found according to gender (χ^2^(1) = 15.34, *p* < 0.001, *φ_c_* = 0.12), marital status (*χ^2^*(4) = 17.84, *p* = 0.001, *φ_c_* = 0.12), occupation (*χ^2^*(3) = 14.50, *p* = 0.002, *φ_c_* = 0.11) and level of education (*χ^2^* (5) = 15.93, *p* = 0.007, *φ_c_* = 0.12). The post hoc analysis revealed that female football members and married/committed members were more likely to be NF; male football fans, who are dating, with elementary education and unemployed were more likely to be FCM. Other column proportions did not differ significantly from each other. An independent sample *t*-test was conducted to test age differences between FCM (*M* ± *SD* = 27.47 ± 10.62) and NF (*M* ± *SD* = 30.64 ± 13.84). The result allowed concluding that the FCM are significantly younger than the NF (*t* (1025, 92) = 4.35, *p* < 0.001). Consequently, H1 (sociodemographic characteristics of football FCM and NF are significantly different) was not rejected. 

A comparative analysis of the groups’ mean values obtained for the BSI dimensions and indexes was carried out to assess H2. The analysis of internal consistency of the BSI factors for the overall sample unveiled acceptable reliability for psychoticism (α = 0.67), and good reliability (0.75 ≤ α ≤ 0.84) for all other dimensions. Composite reliability values of 0.60 to 0.70 are acceptable in exploratory research, as sustained by Hair et al. [49]. Independent samples *t*-tests with Bonferroni correction were applied to study the BSI subscales and indexes values between FCM and NF. Results are shown in Table 3. The BSI dimensions of somatization, anxiety, hostility, paranoid ideation and psychoticism, and the BSI indexes of the FCM group presented significantly higher mean values than the NF group, but with small effect. Therefore, H2 (football FCM present significantly higher BSI values than NF) is not rejected.

To test H3, the normative mean values determined by Canavarro [42] in 2007 for the Portuguese population (Table 1) were compared with those obtained for BSI dimensions and indexes of FCM and NF groups. The results of the independent samples *t*-tests with Bonferroni correction conducted are shown in Table 4.

When compared with the normative values for the general population, the FCM group presented significantly lower mean values (small effect size) for *obsession–compulsion*, and significantly higher mean values for *paranoid ideation* and *PSI* (small effect) and for *hostility* (medium effect). The only non-significant difference found between the means of FCM and the population with emotional disturbance concerns the *hostility* dimension. In the remaining dimensions and indexes, the FCM presented significantly, with large effect, lower mean values than the population with emotional disturbance except for the *paranoid ideation* subscale, where the effect is medium. The NF group showed significantly lower mean values, with large effect size, than the population with emotional disturbance for all BSI subscales and indexes, except for *paranoid ideation*—medium effect—and *hostility*—small effect size. This group also presented significantly, but with small effect size, lower mean values than the general population for *obsession–compulsion*, *interpersonal sensitivity*, *depression, anxiety, GSI* and *TPS*. Specifically, for PSI, when compared with the cut-off point of 1.7, above which there is emotional disturbance [42], results allowed to conclude that the PSI mean value for the NF group was significantly lower (*t* (758) = −8.97, *p* < 0.001), but for the FCM group was not significantly different (*t* (406) = −1.41, *p* = 0.159). Therefore, H3, which states that football FCM and NF present significant differences in the BSI dimensions and indexes values when compared with the normative values of the BSI Portuguese version established by Canavarro [42] in 2007 cannot be rejected.

## 4. Discussion

This study allows us to reach four main results: (1) the sociodemographic profile of the football FCM; (2) the differences between FCM and NF, regarding the BSI values; (3) the differences between the normative values presented by Canavarro [42] in 2007 for the Portuguese population and the values of the FCM and the NF; and (4) the non-psychopathological profile of the NF.

Regarding the four mentioned results above, the following can be stated: 

(1) The football FCM, similarly to the NF, is mostly male, has an affective relationship, does not have children, has secondary education or a high degree, and is employed or is a student. Furthermore, FCM is more likely to be male, dating, unemployed and to have elementary education than the NF. Additionally, FCM is significantly younger than the NF. These results are in agreement with those reported in the literature. In fact, and according to Linden and Linden [31], football fans are traditionally predominantly white, male, and belonging to the working class. Additionally, according to Wakefield and Wann [20] and Rudd [21], highly dysfunctional fans can be characterized as having less education and lower incomes, be younger, male, single, and living in apartments without children at home. Armstrong and Giulianotti [16], Finn and Giulianotti [17], Parry et al. [18] and Shapiro, Ridinger and Trail [19] also found similarities in values and behaviors associated with subcultures of fans, especially regarding the incorporation of traditional western male culture values. 

(2) FCM present significantly higher BSI mean values than NF, in relation to *somatization, anxiety, hostility, paranoid ideation* and *psychoticism* subscales. With respect to the BSI indexes, the FCM also presented significantly higher mean values than NF in all indexes, and their PSI mean value is not significantly different from the cut-off point (1.7) above which there is emotional disturbance [42]. This dichotomy between FCM and NF seems to overlap with the distinction made by Wakefield and Wann [20] and Rudd [21] when these authors distinguished two groups of highly identified sports fans: those with low dysfunctionality and those presenting a high level of dysfunctionality. Moreover, and according to Martins and Martins [50], violence associated with football is mostly the responsibility of organized football fan clubs, although some non-fan club members may also be responsible for some of that violence.

(3) The FCM presented significantly lower mean values for *obsession–compulsion* and significantly higher mean values for *paranoid ideation, hostility* and *PSI* than the general Portuguese population; additionally, it presented significantly lower mean values in all dimensions and indexes than the population with emotional disturbance, with exception of the *hostility* subscale, where the difference is not significant. The NF group has significantly lower values than the general population for *obsession–compulsion*, *interpersonal sensitivity*, *depression, anxiety, GSI* and *TPS*; additionally, it has significantly lower mean values than the population with emotional disturbance for all BSI subscales and indexes. The presence of clinical hostility in NF club members may be justified by the perspective of Gantz [15], an author for whom the reasons for football fans to develop dysfunctional behavior may lie in the fact that these fans present a predisposition to violent physical confrontations. Yu and Wang [51] and Kerr, Wilson, Nakamura and Sudo [52] also suggested that the fans of a losing team scored significantly higher in annoyance, anger, moodiness, humiliation and resentment and significantly lower in relaxation. In accordance with this, Theodorakis, Wann, Al-Emadi, Lianopoulos and Foudouki [53] and Dimmock and Grove [54] consider that highly identified sports fans feel less control over their behavior at games than fans with a moderate or low identification. Hirt et al. [24] and Hoogland et al. [25] also consider that strongly identified sports fans respond to the performance of the team as if team success were a personal success and the failure of the team were a personal failure. When considering Pereira [29] and Belli, Gürbüz and Biricik [30], for whom the most common reasons to be a football fan are the membership of the group, aesthetic, entertainment and eustress, it may be suggested that the dysfunctional behaviors of FCM can be related to motivation to relieve stress due to several factors beyond the football game. One of the most interesting results of this study concerns the fact that when comparing the normative values of the general population of the Portuguese version of the BSI with those of the FCM group, the latter presents significantly lower values for *obsession–compulsion*. Obsession–compulsions is related with the need to perform some behaviors to alleviate anxiety. In fans, this anxiety (worry and fear) is not present. This absence of fear may explain the violent behavior that these FCM often present [55]. According to Daniel and colleagues [38], football fans show less activation in brain regions involved in pain perception and empathy (anterior cingulate cortex, fusiform gyrus, insula and temporal pole) than NF.

(4) Another finding of this study is related to the fact that NF present lower values than the general population; this happens in most of the subscales and BSI indexes. These data seem to suggest that NF are healthier from a psychological point of view than the Portuguese general population. Additionally, they seem to point to a protective effect of the role of NF.

The results of this study support the findings of Russel and Goldstein [56] that reported that football fans scored higher in a general measure of psychopathy than NF. According to Fanti et al. [57], callous-unemotional traits and impulsive-irresponsibility feature the football fan. In addition, Carriedo, Cecchini and González [58] revealed that the frequency of watching football matches was positively associated with low levels of moral behavior and high aggression levels. Additionally, Larkin and Fink [59] found a moderating role of collective narcissism on the relationship between team identification and both dysfunctional fandom and aggression. According to the social problem theory of Ward Jr [60] and King [61], the identification of values, from a clinical point of view, regarding the *paranoid ideation* and *hostility* of football fans with dysfunctional behavior, should make the scientific community rethink theories that are generally focused on group explanations, rather than on individual explanations that are obviously enhanced by group behavior.

This study intends to be innovative when addressing a subject that is generally studied by sociology [62] and not by psychology, and is centered on the group and not on the individual. At the same time, the possible use of these results and other subsequent confirmatory studies may serve to protect all of those who attend football events. However, the representativeness of the sample, limited to a few National Portuguese football teams, represents a weakness of this study, along with the lack of literature in this area. Further investigations should address a greater number of football teams and use an increased sample dimension.

## Figures and Tables

**Table 1:** Normative values of Brief Symptom Inventory (BSI) subscales and indexes of the Portuguese version of the BSI by Canavarro [42] in 2007.

BSI	General Population (*n* = 404)		Emotional Disturbances Population (*n* = 147)	
	*M*	*DP*	*M*	*DP*
Somatization	0.573	0.916	1.355	1.004
Obsession–compulsion	1.290	0.878	1.924	0.925
Interpersonal sensitivity	0.958	0.727	1.597	1.033
Depression	0.893	0.722	1.828	1.051
Anxiety	0.942	0.766	1.753	0.940
Hostility	0.894	0.784	1.411	0.904
Phobic anxiety	0.418	0.663	1.020	0.929
Paranoid ideation	1.063	0.789	1.532	0.850
Psychoticism	0.668	0.614	1.403	0.825
General Symptom Index (GSI)	0.835	0.480	1.430	0.705
Total Positive Symptom (TPS)	26.993	11.724	37.349	12.166
Positive Symptom Index (PSI)	1.561	0.385	2.111	0.595

Source: Canavarro [42].

**Table 2:** Sociodemographic characterization of the overall sample and subsamples.

	Total Sample (*N* = 1166)	Fan Club Members Group (*n* = 407)	Non-Fan Club Members Group (*n* = 759)
*Age^a^*	24 (20, 36)	23 (20, 31)	24 (20, 39)
*Gender (%)*			
Female	37	29	41
Male	**63**	**71**	**59**
*Marital Status (%)*			
Single	46	48	45
Dating	**24**	**28**	**21**
Married/Committed	**24**	**17**	**28**
Divorced/Separated	5	5	5
Widow	1	1	1
*Children (%)*			
Yes	29	26	30
No	**71**	**74**	**70**
*Qualifications (%)*			
Basic education (4 years)	1	1	1
Elementary education (5 years)	10	14	8
Secondary education (3 years)	**44**	**44**	**44**
Graduates	**34**	**33**	**35**
MSc Degree	9	8	10
PhD degree	2	1	2
*Occupation (%)*			
Employed	**48**	**46**	**49**
Unemployed	7	11	5
Retired	3	2	4
Student	**42**	**41**	**42**

*^a^* Median (25th and 75th percentiles); Variables in italic; Higher percentages in bold.

**Table 3:** Descriptive statistics, Cronbach’s alpha (α) and *t*-tests results with Bonferroni correction of BSI dimensions and indexes.

	Total Sample (*N* = 1166)			Fan Club Members (*N* = 407)		Non-Fan Club Members (*N* = 759)		*t-Test*			
BSI	M	SD	*α*	*M*	*SD*	*M*	*SD*	*t-value*	*df*	*p^(1)^*	*d*
*Somatization*	0.47	0.54	0.80	0.53	0.56	0.43	0.53	−3.06	1164	**0.024**	0.187
*Obsession–compulsion*	1.10	0.67	0.75	1.13	0.67	1.08	0.67	−1.26	1164	1	0.077
*Interpersonal sensitivity*	0.78	0.76	0.80	0.86	0.81	0.74	0.73	−2.59	764.615	0.120	0.161
*Depression*	0.77	0.71	0.84	0.82	0.74	0.75	0.70	−1.69	1164	1	0.103
*Anxiety*	0.75	0.60	0.75	0.82	0.62	0.71	0.59	−2.88	1164	**0.048**	0.176
*Hostility*	1.14	0.79	0.79	1.38	0.81	1.02	0.74	−7.55	772.770	**<0.001**	0.470
*Phobic anxiety*	0.38	0.57	0.79	0.39	0.60	0.38	0.56	−0.28	1164	1	0.017
*Paranoid ideation*	1.17	0.82	0.79	1.29	0.84	1.10	0.80	−3.65	794.894	**<0.001**	0.226
*Psychoticism*	0.65	0.63	0.67	0.74	0.64	0.60	0.62	−3.46	1164	**0.012**	0.211
*GSI*	0.80	0.53	-	0.88	0.54	0.75	0.52	−3.82	1164	**<0.001**	0.233
*TPS*	25.36	12.17	-	26.95	12.19	24.51	12.09	−3.28	1164	**0.012**	0.201
*PSI*	1.59	0.45	-	1.67	0.46	1.56	0.44	−4.06	1164	**<0.001**	0.248

(1) Bonferroni-corrected *p*-value; BSI dimensions in italic.

**Table 4:** Independent samples *t*-tests results with Bonferroni correction for differences between: the mean values of BSI dimensions and indexes and the normative values of the BSI Portuguese version [42] in 2007.

BSI	General Population (*N* = 404)								Population With Emotional Disturbance (*N* = 147)							
	Fan Club Members (*N* = 407)				Non-Fan Club Members (*N* = 759)				Fan Club Members (*N* = 407)				Non-Fan Club Members (*N* = 759)			
	*t-value*	*df*	*p* *^(1)^*	*d*	*t-value*	*df*	*p* *^(1)^*	*d*	*t-value*	*df*	*p* *^(1)^*	*d*	*t-value*	*df*	*p* *^(1)^*	*d*
Somatization	0.75	664.697	1	−0.053	2.82	548.851	0.060	−0.186	9.54	180.116	**<0.001**	1.024	10.98	162.225	**<0.001**	1.162
Obsession–compulsion	2.91	752.801	**0.** **044**	−0.205	4.19	656.812	**<0.001**	−0.269	9.57	203.300	**<0.001**	0.987	10.56	176.484	**<0.001**	1.049
Interpersonal sensitivity	1.85	801.574	0.781	−0.130	4.89	1161	**<0.001**	−0.301	7.88	214.656	**<0.001**	0.799	9.66	175.469	**<0.001**	0.963
Depression	1.37	809	1	−0.096	3.22	1161	**0.** **016**	−0.197	10.74	200.758	**<0.001**	1.112	11.97	171.971	**<0.001**	1.210
Anxiety	2.44	771.464	0.177	−0.172	5.24	659.972	**<0.001**	−0.335	11.15	193.765	**<0.001**	1.168	12.93	168.940	**<0.001**	1.325
Hostility	−8.77	809	**<0.001**	0.616	−2.80	1161	0.063	0.171	0.37	552	1	0.035	4.94	186.235	**<0.001**	0.473
Phobic anxiety	0.68	809	1	−0.048	1.04	715.202	1	−0.065	7.66	191.640	**<0.001**	0.805	8.07	167.067	**<0.001**	0.834
Paranoid ideation	−4.02	806.657	**<0.001**	0.282	−0.82	831.331	1	0.050	2.96	552	**0.** **039**	0.284	5.90	904	**<0.001**	0.521
Psychoticism	−1.59	807.670	1	0.112	1.84	1161	0.786	−0.114	8.75	211.925	**<0.001**	0.891	11.10	178.859	**<0.001**	1.092
GSI	−0.55	809	1	0.039	3.50	1161	**0.006**	−0.220	8.54	210.082	**<0.001**	0.872	11.05	177.542	**<0.001**	1.093
TPS	0.05	809	1	−0.003	3.37	1161	**0.** **009**	−0.208	8.87	552	**<0.001**	0.854	11.77	904	**<0.001**	1.059
PSI	−3.67	789.711	**0.003**	0.258	0.00	911.590	1	0.000	8.08	211.148	**<0.001**	0.823	10.58	177.626	**<0.001**	1.045

(1) Bonferroni-corrected *p*-value.
